# The changing epidemiological pattern of Dengue in Swat, Khyber Pakhtunkhwa

**DOI:** 10.1371/journal.pone.0195706

**Published:** 2018-04-24

**Authors:** Jehangir Khan, Abdul Ghaffar, Shujaat Ali Khan

**Affiliations:** 1 Department of Parasitology, Zhongshan School of Medicine, Sun Yat-sen University, Guangzhou, Guangdong, China; 2 Sun Yat-sen University-Michigan State University Joint Center of Vector Control for Tropical Diseases, Zhongshan School of Medicine, Sun Yat-sen University, Guangzhou, Guangdong, China; 3 Department of Zoology, Abdul Wali Khan University Mardan (AWKUM), Khyber Pakhtunkhwa, Pakistan; 4 Department of Meteorology, COMSATS Institute of Information Technology Islamabad, Islamabad, Pakistan; 5 Department of Biotechnology & Bioinformatics, Islamic International University Islamabad, Pakistan; University of Malaya, MALAYSIA

## Abstract

**Background:**

Pakistan is suffering from dengue fever since 1994. In the country, major dengue outbreaks have been documented in 2010, 2011 and 2013 in Punjab (Lahore) and Sindh (Karachi) Provinces. District Swat was hit for the first time by dengue virus in 2013, claiming 57 deaths and 8000 morbidities. The molecular and entomological aspects along with the ecological and social context of the dengue outbreaks were investigated in this study.

**Method:**

In addition to entomological survey, the data collected from patients' files (Saidu Sharif Teaching Hospital, Swat) and by directly questioning the patients (field data) was analyzed for epidemiological trends, molecular identification (RT-PCR based serotyping of DENV), clinical profile, socioeconomic status (SES) and demographic features.

**Results:**

The peak prevalence of dengue was documented in September (56% in 2013, 38% in 2014) and October (33% in 2013, 24% in 2014), whereas in 2015, in October (54.5%) and November (30.5%). The serotype 3 (≤60%) and serotype 2 (≤40%) were found dominant in the area. Among the reported patients (5513), 69% were males and 31% females. Majority of them were found in the age of 14–30 years (52.5%) as compared to 31–60 years (34.5%) (Chi-square: 3219.463, p-value: 0.00). About 63% cases reported in low SES. Among the different categories of government employees affected with dengue, majority (4%) were belonging to health department (Chi-square: 4541.011, p-value: 0.00). Similarly, dengue targeted the dwellers living in multiple-storey houses (65%) as compared to those in the single-storeyed houses (35%) (Chi-square: 495.630, p-value: 0.00). The overall death toll observed was of 57 persons. Dengue prevailed more (38.4%) among low qualified individuals as compared to high qualified (11.5%) (Chi-square: 884.315, p-value: 0.00).

**Conclusion:**

Our analysis indicated a decrease in the epidemiological trend of dengue (now) in the area, though initially it was observed affecting all types of communities on a larger scale. However, the DENV-2 and DENV-3 were dominantly circulating in the area and the prevalence (with usual peaks in post-monsoon) found high in males, illiterate (less educated) individuals and in those with low SES. Urbanization, infected human travelling, climate change, socioeconomic, sociodemographic as well as the wide range adaptation of vector mosquitoes, altogether, are the important factors playing role in the expansion of dengue. Further studies are needed to determine the association of these variables with the dengue spread in the area.

## Background

Dengue is a mosquito borne emerging infectious disease. The responsible virus is known as dengue virus (DENV) which is a single stranded positive-sense RNA belonging to genus *Flavivirus* and family Flaviviridae [1,2]. The virus has different (genetically) but antigenically related four serotypes (DENV-1, DENV-2, DENV-3 and DENV-4) [3–8]. Studies have reported approximately 50 to 100 million infections each year leading to 500,000 hospitalizations and 20,000 deaths [1,2,9,10]. Dengue is endemic in Pakistan with its usual peaks in the post-monsoon period [11,12]. Pakistan suffered 1^st^ outbreak of dengue in 1994 in Karachi [9]. Thereafter, numerous outbreaks have been reported from many parts of the country especially Karachi, Quetta and Lahore. The two recent largest dengue pandemics were recorded first time in Lahore (2011) with 22562 cases and 363 fatalities, and for the second time in Swat (2013) causing 8343 morbidities along with 57 deaths. The predominant circulating virus serotypes reported were DENV-2, 3 & 4 in Punjab (2011), and DENV-2 & 3 in Khyber Pakhtunkhwa (2013) [1,2,13].

Dengue vectors are reported before and after the creation of Pakistan [9,14,15]. The *Aedes aegypti* (primary) and *Aedes albopictus* (secondary), the day biting vector mosquitoes, are responsible for the transmission of all DENV serotypes [10]. These mosquitoes ingest the viremic blood from dengue patients and transfer virus to healthy individuals by releasing its saliva (containing DENV) at the time of bite. After an incubation period of 4–14 days, symptomatic patients typically experience a self-limiting febrile illness with one or more of the symptoms (high fever of up to 40°C, headache, retro-orbital pain, nausea/vomiting, myalgia, arthralgia, rashes) [1,2,16]. Though multiple reports have already been published on epidemiology of dengue, this research was specifically designed to observe the demographic, socioeconomic, entomological, molecular, clinical and epidemiological features of dengue in Swat, KPK.

## Methods and materials

### Description of study area

**Fig 1:**
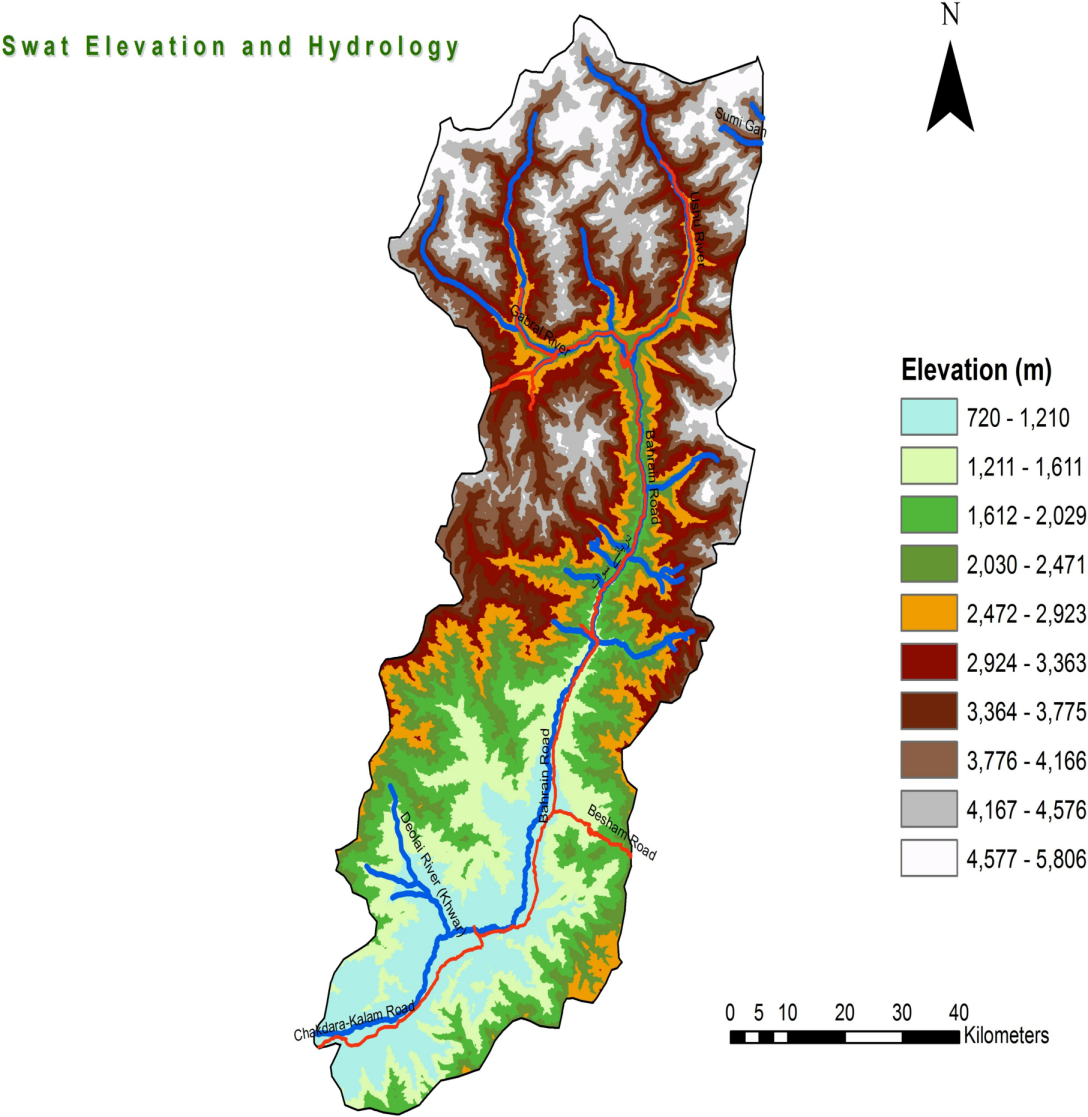
Map of Swat, Khyber Pakhtunkhwa.

Swat (5367 km^2^) (34° 36ʹ 56ʺ -36° 02ʹ 54ʺ N & 71° 42ʹ 30ʺ-072° 07ʹ 05ʺ E) has somewhat warm and humid climate with short and moderate summers; temperature rarely rises above 37°C. The annual rainfall averages around 33 inches with about 17 inches during June-September. The human population of Swat is about 2.31 million. With high mountains, green meadows, and clear lakes, it is a place of great natural beauty and is a popular spot for tourists (Fig 1). This district is bordered by Shangla, Buner, Dir (Malakand) and Chitral. It has numerous habitats for mosquito breeding [9].

### Study type and sampling strategies

This research (June 2013 to November 2015) is descriptive (epidemiological, clinical, and entomological based analysis of the dengue outbreak) and analytical (identification of DENV in serum and mosquito) in nature. It was aimed to identify the circulating serotypes, and to investigate the demographic, molecular, clinical and epidemiological features of dengue patients admitted in Saidu Sharif Teaching Hospital, Swat (STHS).

#### Ethics approval and consent to participate

The study and related protocols were designed according to the national ethical legislative rules and approved by the Local Ethic Committee of Zoology Department, Abdul Wali Khan University Mardan, Buner Campus. All samples were collected after written consent of the individuals (blood donors) according to the updated version of the declaration of Helsinki [17].

#### Collection of clinical data

The data of dengue (laboratory confirmed) patients admitted in STHS during 2013–2015 was collected with the courtesy of Medical Superintendents (MS) of the hospital. STHS is considered the largest hospital with dengue emergency units where a large number of patients come for treatment from Swat.

### Field work

#### Sample collection and an observational survey

Some blood sampling was done during acute phase (i.e 0–5 days) of illness, about 4 ml blood was collected (in EDTA tubes) from all admitted dengue patients (laboratory confirmed) around the year. After serum isolation, the sample was processed for molecular identification of the DENV circulating during the period (2013–2015). The dengue fever case was defined as feverish illness (38.5°C) and having body/joint pains and one of the following: headache, rash, nausea/vomiting, or hemorrhagic manifestations. DF, DHF, and DSS were recognized according to the defined criteria of WHO [9]. A questionnaire comprising multiple questions (history, clinical symptoms, and other information about the disease along with the diagnostic results) was circulated and filled by each dengue patients. Moreover, the patients were contacted for information (concern) missing in the hospital data (their addresses already existed in individual’s record). The exclusion criteria included individuals with confirmed reports of malaria, cancer, tuberculosis, HIV, and bacterial and other parasitic illness.

#### Collection and identification of mosquito

The basic unit for sampling was water-holding containers in the houses of dengue patients according to the protocols [9]. The adult mosquito collection was carried out with the help of back pack aspirator, while that of larvae with iron dipper and sucker machine [9]. The specimens were preserved in 70% formalin and identified to species level using the Leopoldo (2004) key [18]. As one of our previous studies regarding the entomological investigation of dengue epidemics (and the effect of local environment on vector mosquito density) in Swat has already been published [9], therefore this study does not concentrate on detail of that aspect. Subsequently, the molecular identification of dengue virus in vector mosquitoes is carried out.

### RNA extraction

RNA was extracted from samples with Favorgine RNA extraction kit (CAT# FAVNKOO1-2) according to the instructions of manufacturer. The RNA was then processed to characterize the circulating serotypes using DENV type specific primers (TS1–TS4, plus D1) using RT-PCR [1,2,9,19]. The negative and positive controls (DENV-1, 2, 3 and 4) were equally considered. Bands of different amplified products were visualized in 2% agarose gels stained with Gel Red (Biotium Inc., USA).

### Data analysis

The data was analyzed; 1) year wise (2013, 2014 and 2015), 2) age wise (1–13, 14–30, 31–60, >60), 3) sex wise (male and female), 4) education wise (primary, middle, bachelor and high class), 5) employment (education, health and administration), 6) socioeconomically (5000–1000, 10000–20000, 20000–35000 and >36000 PKR), 7) sociodemographically (rural and urban area, living in double/single storey house, and etc.), 8) epidemiology and clinical features of the dengue patients so as to observe the critical period of dengue spread and to look for an association of vector mosquito density and the rate of hospitalization of dengue patients, also to recognize the circulating serotypes of DENV among the vector mosquitoes (in adults and larvae) and blood serum. At the end to observe any other possible factors responsible for the spread of dengue in the district. The statistical analysis of the data was performed using software (SPSS).

## Results

### Identification of DENV in blood samples

We processed 220 blood samples to recognize the type of DENV (circulating among the human population) and its relative abundance. The analysis of the blood samples is; among 100 blood samples (collected in 2013), only 40 samples were positive for DENV-2 and 55 samples for DENV-3, while 5 samples indicated mixed infection of these two serotypes. Similarly, of the 100 samples collected in 2014, only 34 were found positive for DENV-2, 65 for DENV-3 and a single sample reported mixed infection. Likewise, out of 20 samples collected in 2015, just 8 were positive for DENV-2 and 12 for DENV-3 (Table 1). The DENV-3 remained dominant as compared to DENV-2 (Table 1).

**Table 1:** Serotypes distribution (PCR based results) in mosquitoes and blood.

**Molecular study of mosquitoes (adults & larvae) collected in 2013**								
Mature/ Immature	Species	No. of Pools	+ Pools	DENV-1	DENV-2	DENV-3	DENV-4	Mix *
Adults	*Ae*. *aegypti*	10	8	-	3	4	-	1
	*Ae*. *albopictus*	7	4	-	2	2	-	-
Larvae	*Ae*. *aegypti*	7	6	-	2	4	-	-
	*Ae*. *albopictus*	6	5	-	2	3	-	
**Total**		**30**	**23 (76.7%)**	**-**	**9 (39%)**	**13(56.5%)**	**-**	**1(4%)**
**Serological study of samples collected in 2013**								
**Blood**				-	**+**	**+**	-	**+**
**No. of samples**				-	**40 (40%)**	**55 (55%)**	-	**5 (5%)**
**Molecular study of mosquitoes (adults & larvae) collected in 2014**								
Adults	*Ae*. *aegypti*	6	4	-	1	3	-	-
	*Ae*. *albopictus*	4	3	-	1	1	-	1
Larvae	*Ae*. *aegypti*	4	3	-	1	2	-	-
	*Ae*. *albopictus*	4	2	-	—	2	-	-
**Total**		**18**	**12 (66.7%)**		**3 (25%)**	**8 (44.4%)**		**1 (8.3%)**
**Serological study of samples collected in 2014**								
**Blood**				-	**+**	**+**	-	+
**No. of blood Samples**				-	**34 (34%)**	**65 (65%)**	-	**1 (5%)**
**Molecular study of mosquitoes (adults & larvae) collected in 2015**								
Adults	*Ae*. *aegypti*	5	2	-	-	2	-	-
	*Ae*. *albopictus*	4	1	-	1	-	-	-
Larvae	*Ae*. *aegypti*	3	1	-	1	-	-	-
	*Ae*. *albopictus*	3	1	-	-	1	-	-
**Total**		**15**	**5 (33.3%)**		**2 (40%)**	**3 (60%)**	-	-
**Serological study of samples collected in 2015**								
**Blood**				-	**+**	**+**	-	-
**No. of blood Samples**				-	**8 (40%)**	**12 (60%)**	-	-

Mix* Mix infection

### Identification of DENV in mosquito pools

All the adult and larval mosquitoes (*Aedes aegypti* and *Aedes albopictus*) were processed into pools (separately), each pool consists 30 specimens. A total of 40 (n = 1200) out of 63 (n = 1890) mosquito pools were positive for DENV. In 2013 sampling (30 pools: 17 adult pools and 13 larval pools), only 23 pools were positive for DENV (Table 1). Out of these 23 pools, 9 were positive for DENV-2, while 13 for DENV-3, and a single pool was observed to have mixed infection of these two serotypes (Table 1).

Out of 18 pools (sampling in 2014), only 12 pools were positive in which three pools indicated DENV-2 and eight pools mentioned DENV-3, while a single pool was positive for mixed infection (Table 1). Similarly, in 2015 dengue outbreak, we collected and analyzed 15 pools in which five were positive. Out of these five pools, two were identified as DENV-2 and three pools as DENV-3 (Table1). The sampling of mosquito was significantly dependent on the availability of mosquitoes in each area. The collected mosquitoes (larvae and adults) were processed irrespective of the sex. The presence of DENV in the larvae confirms its transovarial transmission among the vector mosquitoes and is a cause of its dissemination. Moreover, the MIR values in our experiment may, in part, be higher because of the greater sensitivity of our virus detection method, RT-PCR, compared with other methods of screening and sample sizes. However, the sequencing and phylogenetic studies of the circulating serotypes in the bordering areas of Swat (Malakand region) has already been published [20–22]. These reports confirm the serotypes we detected in the area. We observed an indispensable association between density of vector mosquito and the rate of hospitalization of dengue patients (a minor observation).

### Epidemiological features

The data analysis resulted in a total of 59765 dengue cases along with 57 deaths reported during 2013, whereas only 5513 patients of them got admitted in the hospital (STHS). In 2014, about 500 dengue cases were reported but only 290 patients were admitted with no death incidence. Till the last day of November 2015, a total of 33 out of 265 dengue patients were admitted in the hospital (Table 2). The percentage analysis of dengue patients showed a declining pattern of dengue from 94% (2013) to 5.4% (2014) and 0.6% in 2015 (Table 2).

**Table 2:** Month, sex and area wise distribution of dengue patients.

		**Total admitted Patients (%)**			**Sex wise deaths**			
**Year**	**Total (OPD)**	**n (%)**	**Sex wise distribution**		**♂**		**♀**	
2013	59000	5190 (94)	♂ (%)	33 (58%)	33 (58%)		24 (42%)	
			3604 (70)	1586 (30)				
2014	500	290 (5.4)	175 (60)	115 (40)	0			
2015	265	33 (0.6)	27 (82)	6 (18)	0			
Total	**59765**	**5513**	**3806** (69)	**1707 (31)**	**57**			
**Month wise distribution of Dengue incidences in Swat during 2013–2015**								
**Year (2013)**		**Year (2014)**		**Year (2015)**				
August	464 (9%)	August	65 (22.5%)	August	0 (0%)			
September	2916 (56%)	September	110 (38%)	September	5 (15%)			
October	1710 (33%)	October	70 (24%)	October	18 (54.5%)			
November	100 (2%)	November	45 (15.5%)	November	10 (30.5%)			
**Total**	**5190**	**Total**	**290**	**Total**	**33**			
**Most affected Areas in Swat during 2013**								
Mingora,Saidu	Spal Bandai, Charbagh, Khwazakhela	Madyan, Kalam,Bahrain, Mian dam	Barikot, Udigram, Balogram	Qambar,Rahimabad	Manglawar,Banjot	Kabal,Matta	Out district	**Total**
**3596(69.3%)**	**230 (4.4%)**	**104(2%)**	**194(3.7%)**	**414 (8%)**	**138 (2.7%)**	**205(3.9%)**	**311(6%)**	**5190**
**Most affected Areas in Swat during 2014**								
Skha Chena, Amankot		Sethi,Amankot	Faizabad	Saidu Sharif	Out district			**Total**
**56 (19.5%)**		**42 (14.5%)**	**84(29%)**	**44 (15%)**	**64 (22%)**			**290**
**Most affected Areas in Swat during 2015**								
Mingora & Saidu		Batkhela		Kohistan	Out district			**Total**
**21 (64%)**		**3 (9%)**		**3 (9%)**	**6 (18%)**			**33**

### Demographic patterns

Out of 5513 admitted patients, 3806 (69%) were males and 1707 (31%) females (Table 2). Majority of the patients (2727) were in the age of 14–30 years (Chi square: 3219.463, p: 0.00) followed by 31–60 years (1792). Least number of cases reported were in the age >60 years (3.8%) (Table 3). Similarly, the data analysis showed less (primary) educated individuals suffered more (38.4%) (Chi square: 884.315, p: 0.00) than highly educated (11.5%) (Table 3). However, no significant difference was observed among the age, sex and socioeconomic profiles of dengue patients in the three years journey of dengue in Swat.

**Table 3:** Age, education and employment record of dengue patients and their SES.

Age group	**Frequency**	**Test statistics**	
1–13	474	Chi-Square	3219.463^a^
14–30	2727	Degree of Freedom	3
31–60	1792	Asymptotic Significant value	.000
Above 60	197		
Total	5190		
a. 0 cells (.0%) have expected frequencies less than 5. The minimum expected cell frequency is 1297.5.			
**Educational profile of the patients**			
Primary	2115	Chi-Square	884.315^a^
Middle	1627	Degree of Freedom	3
Bachelor	1139	Asymptotic Significant value	.000
Higher	632		
Total	5513		
a. 0 cells (.0%) have expected frequencies less than 5. The minimum expected cell frequency is 1378.3.			
**Basic Pay Scale (BPS: 1–20) wise distribution of Patients**			
1–10	103	Chi-Square	32.844^a^
11–16	57	Degree of Freedom	2
17–20	39	Asymptotic Significant value	.000
Total	199		
a. 0 cells (.0%) have expected frequencies less than 5. The minimum expected cell frequency is 66.3.			
**Socioeconomic status of the patients (monthly income in Pak rupees)**			
5000–10000	3475	Chi-Square	4541.011^a^
10001–20000	1168	Degree of Freedom	3
20001–35000	574	Asymptotic Significant value	.000
above 35000	296		
Total	5513		
a. 0 cells (.0%) have expected frequencies less than 5. The minimum expected cell frequency is 1378.3.			
**Departmental wise distribution of Dengue patients**			
Education	111	Chi-Square	4541.011^a^
Administration	181	Degree of Freedom	3
Health	208	Asymptotic Significant value	.000
Public	5013		
Total	5513		
a. 0 cells (.0%) have expected frequencies less than 5. The minimum expected cell frequency is 1378.3.			
**Area wise distribution of patients**			
Rural	1654	Chi-Square	881.920^a^
Urban	3859	Degree of Freedom	1
Total	5513	Asymptotic Significant value	.000
a. 0 cells (.0%) have expected frequencies less than 5. The minimum expected cell frequency is 2756.5.			
**Building type**			
Double/Multiple Storey	3583	Chi-Square	495.630^a^
Single Storey	1930	Degree of Freedom	1
Total	5513	Asymptotic Significant value	.000
a. 0 cells (.0%) have expected frequencies less than 5. The minimum expected cell frequency is 2756.5.			

Furthermore, the data analysis also resulted in minimum (2%) admissions (hospitalization) of dengue patients in the month of November while maximum in September (56%) in 2013. In 2014, maximum hospitalization was recorded again in September (38%) and lowest (15.5%) in November, whereas in 2015, majority (54.5%) of the patients were admitted in October (Table 2).

### Socioeconomic trend of dengue

According to the census report of 2017, the population of Swat is 2.31 million with the total area 537 Km^2^. Almost the whole district (Swat) was hit by dengue, but the residence based data analysis reported more prevalence among patients (70%) (Chi square: 881.920, p: 0.00) from urban parts (Mingora, Saidu Sharif, Gulkada, Faizabad, Amankot, Rahimabad, Qambar, Malookabad, Manglawar and Takhtaband) of the area as compared to rural (30%) (Kabal, Sirsinai, Hazara, Aligrama, Barabandai, Kozabandai, Dherai, Matta), the details of rural and urban areas (Swat) have already been published [9] (Tables 2 and 3).

One of the important objectives of this study is to find out the disease as most common in poor communities. Three categories of communities were made on the basis of monthly income (Pakistani currency: PKR) (Table 3) according to the protocols [23–25]. The individuals from poor (500–10000 pkr/month) communities were mostly targeted (65%) (Chi square: 4541.011, p: 0.00) than middle class (21.4%) communities, whereas, those having monthly income higher than 36000 pkr/month were less in numbers (5.4%) (Table 3). Dengue epidemics in Swat were observed at a broader scale affecting the government employees of different basic pay scale (BPS) from 1 to 20 (Grades) belonging to many departments (education, administration and health) during the time in question (Table 3). Among these, the employees having BPS 1–10 were more in numbers (51.8%) (Chi square: 32.844, p: 0.00) as compared to those having BPS 17 to 20 (19.4%). About 4% (Chi square: 4541.011, p: 0.00) of the dengue cases were belonging to health department (mostly lower staff) and 2% from education (Table 3). The record analysis also showed that only eight dengue patients had travelling history from Malaysia and Lahore where recently the dengue epidemics were reported. Moreover, it was also found that the dwellers living in multiple-storey buildings suffered more (65%) (Chi square: 495.630, p: 0.00) as compared to those in single storey buildings (35%) (Table 3).

### Clinical profile of the dengue patients

Among the severe signs of dengue, increased hematocrit (concurrent with decreased platelet count) was common in 85% of the patients followed by liver enlargement (>2cm) in 70% patients, while the less common (35%) symptom was persistent vomiting (Table 4).

**Table 4:** The laboratory findings of dengue patients (2013–2015).

**Symptoms developed by the dengue patients at the time of death (n∙ = 57)**								
DF with intestinal obstruction	Renal failure With DHF	DSS* with aspiration	DSS	DHF°	Glumerolo Nephritis & Pulmonary Edema With DHF	DF with Bronchial asthma	DHF with pleural effusion	DF with left arm cellulites
3	2	2	36	9	1	1	2	1
• DSS* Dengue shock syndrome • DHF° Dengue hemorrhagic fever • n number of deaths								
**Platelets counts in the dengue patients**								
Patients with PLT < 100000		Patients with PLT < 50000			Patients with PLT < 20000		**Total**	
1579		632			153		**2364**	
**2013 Data about Patients**								
**Travel history**	**NSI +**	**IgG/IgM+**		**Discharged**	LAMA*	**Referred**	**Local patients**	**Outdoor patients**
Lahore, Malaysia (n = 5)	1786	728		5104	57	29	4790 (92.3%)	400 (7.7%)
**2014 Data about Patients**								
Lahore, Malaysia (n = 3)	287	27		287	NA	0	226 (78%)	64 (22%)
**2015 Data about Patients**								
NA	33	—		30	2	1	27 (81.8%)	6 (18.2%)

*Leave against medical advice

## Discussion

The earlier studies have depicted the occurrence of dengue in multiple cities of the country, but for the first time some socioeconomic factors were taken into account along with the molecular and clinical studies to know the dengue dynamics in Swat. The results of the study indicate that Swat is recovering from its first dengue fever outbreak, with the dropping number of patients from 94% (2013) to 5.4% (2014) and 0.6% (2015). However, dengue on the contrary, is on rise on national level [26,27]. The declining trend of the disease in the area reflects an awareness (to fight with the disease) among the inhabitants about dengue or may be it is because of the development of resistance in individuals against the disease.

The demographic results of this study showed high prevalence (69%) of dengue in males than in females (31%), with age group 14–30 years more suffered. The low occurrence in females may be due to their segregation (to a large extent), maximum stay at home, and proper covering of their bodies by wearing long shirts and head scarp (cultural dress of the area) thereby protecting them from mosquitoes’ bite. The specific age group of young people targeted more is due to their exposure to different environments. This could be another possible reason that in summer the males and children sleep at night without wearing shirts (often) and as a result remain exposed to vector mosquito bite: an observation congruent with [9]. Our findings are in accordance with national and South Asian studies [1, 2, 24–26] but dissimilar to the reports from India and Singapore [24,28].

Moreover, our findings support the hypothesis that communities having better SES enable them to get better education and knowledge, easy access to information about disease and disease outbreak, and hence are less targeted by dengue [25, 29–32]. On the contrary, higher cases (63%) of dengue were documented in low income communities (earning less than 11000 PKR/month) and having lower SES. Our results are in agreement with the previous reports [29,33–37]. This suggests that efficient programs about awareness (dengue) should be initiated in general populations specifically those with low SES. Analysis of the residential (geographic) record of patients showed that the disease was common in urban dwellers (70%); an observation consistent with the other national studies [1,2,11–12]. However, it may be a limitation of our study to justify the case of a patient who spent most of his/her time in urban areas (may be due to his/her occupation) but had a rural residential address. Rise in urbanization, mingling of people, more travel and trade subsequently may have led to dengue spread in the area [9, 11, 12]. Our results suggest for a study to test the relative association of these factors (i.e. SES, sociodemographic, and travel and trade) with the recent expansion of dengue in the country.

The change in temperature and humidity (ecological conditions) plays a significant role in the survival/breeding of vector mosquitoes and its population density [11–12]. A more recent study [9] has shown that higher temperature (>25°C) produces large number of mosquitoes with frequent blood feeding nature. Also, it is documented [9] that 1°C increase in temperature (above average) may increase the risk of dengue transmission by 1.95 times [9,11,12,28–29]. The peak in dengue cases (hospitalization of the patients) during September-November (2013–2015) in our study may be directly linked to an increased population and repeatedly feeding nature of *Aedes* mosquitoes (due to favorable environment for mosquito) on humans, we are in agreement with many national reports [20–22]. Altogether, these months are suggested to be more critical for the disease to spread therefore, precautionary steps should be adopted during this period. Nonetheless, further studies are suggested to determine the impact of local environment (ecological) on the density of vector mosquitoes and the subsequent spread of dengue.

Identification of DENV in human blood during an outbreak alone may not be much supportive in predicting the dengue outbreaks [9,38]. Identification of DENV in vector (mosquitoes) has offered a trustworthy tool to figure out the prevailing serotype in an area which is helpful in designing most efficient strategies for mosquito control. Also, the surveillance of infected vector mosquitoes will assist to predict the future outbreaks of dengue in an area through the phenomena of minimum infection rates (MIRs) in mosquitoes of that area [9,38–47]. We reported both the species of *Aedes* from Swat. However, *Ae*. *aegypti* was dominant and widely distributed as compared to *Ae*. *albopictus* (Table 1). Similar results have been reported from other cities of Pakistan [38], especially in KPK [1,2,9]. It has also been documented [9] that a single mosquito (infected with DENV) may serve as a reservoir of DENV and its (virus) subsequent transmission among the vector (mosquitoes) as well as humans. The more dengue cases reported among dwellers in multiple storey houses (in our study) may be due to availability of more supportive (breeding) places/habitats for mosquito to breed and bite (i.e. opportunistic) the inhabitants. Our results are congruent with the country wide studies [11,12,20–22]. Early warning system (EWS) about dengue outbreak is extremely important for the control of this disease but vector control measures are generally initiated after the occurrence of viral infection in the human population. The time span for this interval (detection of transovarial dengue virus and indication of clinical symptoms in humans) ranges from 7–41 days and after the clinical symptoms in human blood, the vector control is insufficient in preventing the dengue epidemic [48]. Dengue virus detection in adult mosquito is possible to predict an outbreak six weeks in advance of the occurrence of first human case [49]. After the virus detection in human populations following measure can be suggested i) dengue prevention and control programs should be implemented with more effective way at governmental level ii) research regarding dengue spread should focus on the efficiency, cost effectiveness and sustainable control methods be implemented with the addition of new diagnostic tools and vector control strategies iii) improved clinical management of severe dengue cases should be enhanced iv) public awareness through early warning system (EWS) for the preparedness of community should be implemented v) research on transmission dynamics i-e., virus population structure, climatic covariates, social covariates in addition to urbanization and other land use changes should be critically addressed, vi) operational research including involvement of local population, entomological surveillance, household water management and identification of vulnerable groups with geographical localities be conducted vii) the development of more effective tools to prevent and control outbreaks i-e genetically modified mosquitos (of dengue), wolbachia based approaches, effective drainage system and use of insecticides should be focused.

This study found all samples of blood and 40/63 mosquito pools are positive for dengue virus. The serotype 3 (≤60%) and serotype 2 (≤40%) were found dominant in the area (Table 1). Recent studies have carried out the phylogenetic analysis of the serotypes (DENV-2 and DENV-3) circulating in the bordering areas of Swat (Malakand region) [20–22] which confirm our recognized serotypes (in the study area). The mosquito eggs survive under unfavorable environment even without a vertebrate host for more than a year and thus, transmit dengue from one place to another [9,29,50,51]. Taking together, this suggests that dengue transmission via virus loaded eggs/adults through different means could be one of the possible reasons of sudden dengue epidemics in Swat. Additionally, this area was affected by floods (2010–11) due to heavy rainfall which may have provided suitable habitats for the establishment of Aedes. We are in accordance with [9,20]. Previously, major dengue outbreaks have been reported in cities like Lahore, Faisalabad and Peshawar in 2011–2012 [11, 12]. Also, the circulation of only two serotypes (DENV-2 & DENV-3) in similar passion (of their relative abundances) (Table 1) in Swat, hypothesize this outbreak as the continuation of previous outbreaks reported in Lahore, Faisalabad, Rawalpindi and Peshawar. This hypothesis is supported by the molecular based studies (conducted in the neighboring areas of Swat) [9,20–22]. Our data analysis also revealed the human travelling as a potent source of DENV transmission from endemic areas to non-endemic areas. For instance, eight patients in this study had a travelling history to Malaysia and Lahore, however, the exact situation is not clear about this fact (Table 4). Nevertheless, our observations are consistent with studies conducted in areas (i.e. Malakand) adjacent to Swat [20–22].

Previous reports lend support to our findings that the serotypes circulating in Swat and Malakand regions (Khyber Pakhtunkhwa Province) have genetic ancestry with the existing serotypes in Karachi (Sindh Province) and Lahore (Punjab Province), where the dengue virus was first introduced into the country from India, Malaysia and other countries [9,13–15,20–22,52–54]. This reveals the geographic extension of local strains of serotypes into non endemic areas of the country. In this study, the DENV positive pools of vector mosquitoes were correlated with maximum threat of dengue reported areas, such as Mingora, Saidu Sharif and Faizabad, which are the most populated and tourist’s spots that harbor mosquitoes and thus spread DENV. Based on these results, further study is needed to determine the reasons of dengue invasion to non-endemic areas in the country.

The clinical symptoms also act as an important tool for diagnosing and managing of the disease (DHF) as defined by the WHO [9,19,29]. Our results show that most of the patients were having thrombocytopenia, high fever, vomiting, increased liver enzymes and hematological imbalances. These symptoms have been reported in previous studies [1,2,4,5,9,16,25,29,55–58]. Conclusively, additional research on the disease spread in the country should take in to consideration the rapid diagnostic facilities, recognizing the prevailing serotypes, the correlation of population dynamics of vector mosquitoes and the disease (dengue), the interplay among socioeconomic, sociodemographic, ecological change and the disease spread, implementation of vector control strategies (sustainable), and public awareness on priority.

## References

[1] Khan J, Khan A. Incidence of dengue in 2013: Dengue outbreak in District Swat, Khyber Pakhtunkhwa, Pakistan, Inter J Fauna and Bio Stud. 2015; 2(1): 01–07.

[2] Khan J, Munir W, Khan BT, Ahmad Z, Sham WA, Khan A. Dengue outbreak 2013: Clinical profile of patients presenting at DHQ Burner and THQ Shangla, Khyber Pakhtunkhwa. Biohelikon: Immunity & Diseases. 2015; 3: a11.

[3] Akram M, Fatima Z, Purdy MA, Sue A, Saleem S, Amin I, et al Introduction and evolution of dengue virus type 2 in Pakistan: a phylogeographic analysis. Virol J. 2015; 12:148 doi: 10.1186/s12985-015-0371-8 26395339 10.1186/s12985-015-0371-8 PMC4579586

[4] Ali J. Dengue fever in Pakistan: Challenges, priorities and measures. J Coast Life Medi. 2015; 3(10): 834–837.

[5] Ali J. Dengue fever: symptoms, treatments and prevention; a general perspective. World J Zool. 2015; 10(1): 22–5.

[6] Khan J, Khan I, Ali I, Iqbal A, Salman M. The Role of Vertical Transmission of Dengue Virus among Field-Captured *Ae*. *aegypti* and *Ae*. *albopictus* Mosquitoes in Peshawar, Khyber Pakhtunkhwa, Pakistan. Pakistan J Zool. 2017; 49(3): 777–784.

[7] Khanani MR, Arif A, Shaikh R. Dengue in Pakistan: journey from a disease free to a hyper endemic nation. J Dow Univ Health Sci. 2011; 5(3): 81–4.

[8] Khan E, Hasan R, Mehraj J, Mahmood S. Genetic Diversity of Dengue Virus and Associated Clinical Severity During Periodic Epidemics in South East Asia. Curr Topics in Trop Medic. 2012; 92–109.

[9] Khan J, Khan I, Amin I. The first Comprehensive Entomological, Serological and Molecular Study of 2013 Dengue Outbreak of Swat, Khyber Pakhtunkhwa, Pakistan. PLoS ONE. 2016: 11(2): e0147416 doi: 10.1371/journal.pone.0147416 26848847 10.1371/journal.pone.0147416 PMC4746065

[10] Khan J, Shah M, Khan BT, Naeem M, Ismail M, Abbasi A, et al A survey of adult and larval mosquito fauna in Tehsil Daggar and Gagra of District Buner, Khyber Pakhtunkhwa, Pakistan. Inter J Mosq Res. 2015; 2(3): 170–174.

[11] Khalid B, Ghaffar A. Dengue transmission based on urban environmental gradients in different cities of Pakistan. Int J Biometeorol. 2014; 267–283. doi: 10.1007/s00484-014-0840-6 24817491 10.1007/s00484-014-0840-6

[12] Khalid B, Ghaffar A. Environmental risk factors and hotspot analysis of dengue distribution in Pakistan. Int J Biometeorol. 2015; 1–26. 10.1007/s00484-015-0982-1 25869291

[13] Koo C, Nasir A, Hapuarachchi HC, Lee KS, Hasan Z. Evolution and heterogeneity of multiple serotypes of Dengue virus in Pakistan, 2006–2011. Viro J. 2013; 10: 275. 10.1186/1743-422X-10-275 PMC3844417 24007412

[14] Rasheed SB, Boots M, Frantz AC, Butlin RK. Population structure of the mosquito *Aedes aegypti* (*Stegomyia aegypti*) in Pakistan. Med and Veter Entomo. 2013; 27(4): 430–440. 10.1111/mve.12001 23662926

[15] Rasheed SB, Butlin RK, Boots M. A review of dengue as an emerging disease in Pakistan. Publ Heal. 2013; 127: 11–17. 10.1016/j.puhe.2012.09.006 23219263

[16] Yacoub S, Wills B. Dengue: an update for clinicians working in non-endemic areas. Clinic Medi. 2015; 15(1): 82–5. 10.7861/clinmedicine.15-1-82 PMC4954533 25650206

[17] WMA Declaration of Helsinki—Ethical Principles for Medical Research Involving Human Subjects. 59th WMA General Assembly, Seoul, October 2008.

[18] Leopoldo MR. Pictorial key for the identification of mosquitoes (Diptera: Culicidea) associated with dengue virus transmission. Zootaxa. 2004; 589: Pp 60.

[19] Lanciotti RS, Calisher CH, Gubler DJ, Chang G-J, Vorndam AV. Rapid detection and typing of dengue viruses from clinical samples by using reverse transcriptase-polymerase chain reaction. J of Clin Micro. 1992; 30: 545–551. 10.1128/jcm.30.3.545-551.1992 PMC265106 1372617

[20] Suleman M, Lee HW, Zaidi SSZ, Alam MM, Nisar N, Aamir UB, et al Preliminary Seroepidemiological survey of dengue infections in Pakistan, 2009–2014. J Infec dise Pove. 2017; 6:48: doi: 10.1186/s40249-017-0258-6 28274279 10.1186/s40249-017-0258-6 PMC5343310

[21] Suleman M, Faryala R, Alam MM, Khurshid A, Sharif Angez M, et al Outbreak of dengue virus type-3 in Malakand, Pakistan 2015; A laboratory perspective. Acta Tropica. 2017; 169: 202–206. doi: 10.1016/j.actatropica.2017.02.011 28219668 10.1016/j.actatropica.2017.02.011

[22] Suleman M, Faryal R, Alam MM, Zaidi SSZ. Demographic characteristics of dengue virus outbreaks in Khyber Pakhtunkhwa Province, Pakistan during 2003–2015. Form Med Asso 2017; http://dx.doi.org/10.1016/j.jfma.2017.02.004. 10.1016/j.jfma.2017.02.004 28291567

[23] Khan J, Aslam F, Khan BT, Anjum SI, Faiz-Ur-Rehman, Shams WA, et al A Study of Socio-Economic Status (SES) Associated with Epidemiology of Tuberculosis in General Population of District Buner, Khyber Pakhtunkhwa (KPK), Pakistan. Open Acc Libra J. 2015; 2: e1514.

[24] Khalil MAM, Tan J, Khalil MAU, Awan S, Rangasami M. Predictors of hospital stay and mortality in dengue virus infection-experience from Aga Khan University Hospital Pakistan. BMC Res Notes. 2014; 7: 473 doi: 10.1186/1756-0500-7-473 25064632 10.1186/1756-0500-7-473 PMC4115468

[25] Itrat A, Khan A, Javaid S, Kamal M, Khan H, Javed S, et al Knowledge, Awareness and Practices Regarding Dengue Fever among the Adult Population of Dengue Hit Cosmopolitan. PLoS ONE. 2008; 3(7): e2620 doi: 10.1371/journal.pone.0002620 18612437 10.1371/journal.pone.0002620 PMC2440812

[26] Ali A, Rehman HU, Nisar M, Rafique S, Ali S, Hussain A, et al Seroepidemiology of dengue fever in Khyber Pakhtunkhawa, Pakistan. Inter J Infec Dis. 2013; 17: 518–523. 10.1016/j.ijid.2013.01.007 23523057

[27] Haider Z, Ahmad FZ, Mahmood A, Waseem T, Shafiq I, Raza T, et al Dengue fever in Pakistan: a paradigm shift; changing epidemiology and clinical patterns. Persp in Pub Health. 2015; 20(21). 10.1177/1757913915599019 26342006

[28] Descloux E, Mangeas M, Menkes CE, Lengaigne M, Leroy A, Tahei T, et al Climate-based models for understanding and forecasting dengue epidemics. PLoS Negl Trop Dis. 2012; 6: e1470 doi: 10.1371/journal.pntd.0001470 22348154 10.1371/journal.pntd.0001470 PMC3279338

[29] Raza FA, Rehman Su, Khalid R, Ahmad J, Ashraf S, Iqbal M, et al Demographic and Clinico-Epidemiological Features of Dengue Fever in Faisalabad, Pakistan. PLOS ONE. 2014; 9(3): e89868 doi: 10.1371/journal.pone.0089868 24595236 10.1371/journal.pone.0089868 PMC3940608

[30] Kubik K, Blackwell L, Heit M. Does socioeconomic status explain racial differences in urinary incontinence knowledge? Am J Obstet Gynecol. 2004; 191: 188–193. doi: 10.1016/j.ajog.2004.03.084 15295363 10.1016/j.ajog.2004.03.084

[31] Potvin L, Richard L, Edwards AC. Knowledge of cardiovascular disease risk factors among the Canadian population: relationships with indicators of socioeconomic status. CMAJ. 2000; 162: S5–11. 10813022 PMC1232442

[32] McArthur L, Pena M, Holbert D. Effects of socioeconomic status on the obesity knowledge of adolescents from six Latin American cities. Int J Obes Relat Metab Disord. 2000; 25: 1262–1268. 10.1038/sj.ijo.0801674 11477513

[33] Dhimal M, Aryal KK, Dhimal ML, Gautam I, Singh SP, Bhusal CL, et al Knowledge, Attitude and Practice Regarding Dengue Fever among the Healthy Population of Highland and Lowland Communities in Central Nepal. PLoS ONE. 2014; 9(7): e102028 doi: 10.1371/journal.pone.0102028 25007284 10.1371/journal.pone.0102028 PMC4090170

[34] Cheong YL, Leitao PJ, Lakes T. Assessment of land use factors associated with dengue cases in Malaysia using Boosted Regression Trees. Spati and Spatio-temporal Epidem. 2014; 10: 75–84. 10.1016/j.sste.2014.05.002 25113593

[35] Mayxay M, Cui W, Thammavong S, Khensakhou K, Vongxay V, Inthasoum L, et al Dengue in peri-urban Pak-Ngum district, Vientiane capital of Laos: a community survey on knowledge, attitudes and practices. BMC Pub Health. 2013; 13: 434. 23641953 10.1186/1471-2458-13-434 PMC3645963

[36] Castro M, Sanchez L, Perez D, Sebrango C, Shkedy Z, Stuyft PVD. The Relationship between Economic Status, Knowledge on Dengue, Risk Perceptions and Practices. PLoS ONE. 2013; 8(12): e81875 doi: 10.1371/journal.pone.0081875 24349145 10.1371/journal.pone.0081875 PMC3861357

[37] Stewart-Ibarra AM, Munoz AG, Ryan SJ, Ayala EB, Borbor-Cordova MJ, Finkelstein JL, et al Spatiotemporal clustering, climate periodicity, and social-ecological risk factors for dengue during an outbreak in Machala, Ecuador, in 2010. BMC Infec Diseas. 2014; 14: 610. 10.1186/s12879-014-0610-4 PMC4264610 25420543

[38] Jahan N, Tanveer A, Zafar S, Zaheer A. Entomological Surveillance and Detection of Dengue Viruses in Vector Mosquitoes as an Early Warning Tool for the Control of Dengue in Pakistan. Biologia (Pakistan). 2014; 60(2): 169–176.

[39] Le Goff G, Revollo J, Guerra M, Cruz M, Barja Simon Z, Roca Y, et al Natural vertical transmission of dengue viruses by *Aedes aegypti* in Bolivia. Paras. 2011; 18: 277–80. 10.1051/parasite/2011183277 PMC3671471 21894270

[40] de Figueiredo MLG, de C Gomes A, Amarilla AA, de S Leandro A, de S Orrico A, de Araujo RF, et al Mosquitoes infected with dengue viruses in Brazil. Virol J. 2010; 7: 152–7. doi: 10.1186/1743-422X-7-152 20624314 10.1186/1743-422X-7-152 PMC2913956

[41] Cecilio AB, Campanelli ES, Souza KPR, Figueiredo LB, Resende MC. Natural vertical transmission by *Stegomyia albopicta* as dengue vector in Brazil. Braz J Biol. 2009; 69: 123–7. 19347154 10.1590/s1519-69842009000100015

[42] Angel B, Joshi V. Distribution and seasonality of vertically transmitted dengue viruses in *Aedes* mosquitoes in arid and semi-arid areas of Rajasthan, India. J Vector Borne Dis. 2008; 45: 56–9. 18399318

[43] Vilela APP, Figueiredo LB, dos Santos JR, Eiras AE, Bonjardim CA, Ferreira PCP, et al Dengue virus 3 genotype I in *Aedes aegypti* mosquitoes and eggs, Brazil, 2005–2006. Emerg Infect Dis. 2010; 16: 989–92. doi: 10.3201/eid1606.091000 20507754 10.3201/eid1606.091000 PMC3086226

[44] Mulyatno KC, Yamanaka A, Yotopranoto S, Konishi E. Vertical transmission of dengue virus in *Aedes aegypti* collected in Surabaya, Indonesia, during 2008–2011. Jpn J Infect Dis. 2012; 65: 274–6. 22627316

[45] Espinosa M, Giamperetti S, Abril M, Seijo A. Vertical transmission of dengue virus in *Aedes aegypti* collected in Puerto Iguazu, Misiones, Argentina. Rev Inst Med Trop Sao Paulo. 2014; 56(2): 165–7. doi: 10.1590/S0036-46652014000200013 24626420 10.1590/S0036-46652014000200013 PMC4085839

[46] Adams B, Boots M. How important is vertical transmission in mosquitoes for the persistence of dengue? Insights from a mathematical model. Epidemio. 2010; 2: 1–10. 10.1016/j.epidem.2010.01.001 21352772

[47] Eva A, Buckner, Barry W, Alto, Lounibos LP. Vertical Transmission of Key West Dengue-1 Virus by *Aedes aegypti* and *Aedes albopictus* (Diptera: Culicidae) Mosquitoes from Florida. J Med Entomolo. 2013; 50(6): 1291–1297. 10.1603/me13047 PMC4031614 24843934

[48] Lee HL, Rohani A. Transovarial transmission of dengue virus to *Aedes aegypti* and *Aedes albopictus* in relation to dengue outbreak in an urban area in Malaysia. Dengue Bulletin.2005; 29: 106–210.

[49] Chow VT, Chan YC, Yong R, Lee KM, Lim LK, Chung YK, et al Monitoring of dengue viruses in field-caught *Aedes aegypti* and *Aedes albopictus* mosquitoes by a type specific polymerase chain reaction and cycle sequencing. Am J Trop Med Hyg. 1998; 58(5): 578–586. 9598444 10.4269/ajtmh.1998.58.578

[50] Joshi V, Mourya DT, Sharma RC. Persistence of dengue-3 virus through transovarial transmission passage in successive generations of *Aedes aegypti* mosquitoes. Am J Trop Med Hyg. 2002; 67: 158–161. 12389940 10.4269/ajtmh.2002.67.158

[51] Lequime S, Lambrechts L. Vertical transmission of arboviruses in mosquitoes: A historical perspective. Inf Gene and Evol. 2014; 28: 681–690. 10.1016/j.meegid.2014.07.025 25077992

[52] Ahmad T, Journey of Dengue from swat to Batkhela Malakand Agency, Pakistan. Int J Mic and Alli Sci. 2014; 1(1).

[53] Idrees M, Hussain W, Rehman HU, Tayyab GN, Afzal S, Fatima Z, et al Dengue Virus Serotype 2 (DEN-2): the Causative Agent of 2011-Dengue Epidemic in Pakistan. Am J Biomed Sci. 2012; 4(4): 307–315.

[54] Mukhtar M, Tahir Z, Baloch TM, Mansoord F, Kamran J. Entomological investigations of dengue vectors in epidemic-prone districts of Pakistan during 2006–2010. Deng Bull. 2011; 35.

[55] Zafar H, Bukhari KT, Lodhi GM. Global prevalence of dengue viral infection, its pathogenesis diagnostic and preventive approaches. Asian J Agric Biol. 2013; 1(1): 38–42.

[56] Manzoor F, Farooq H, Kanwal Z, Bibi F. A Study on Dengue Knowledge, Attitude, Practices and their Impact on *Aedes aegypti* Population in Lahore, Pakistan. Pak J life soc. Sci. 2015; 13(3): 145‐152.

[57] Alam I, Hassan S, Alam I, Gul R, Ali F, Ali I, et al PAIgG and PAIgM levels in secondary dengue virus infections lead to thrombocytopenia in patients from KP, Pakistan. Asian Pac J Trop Biomed. 2015; 5(10): 801–805.

[58] Hasan SR, Riaz M, Jafri FA. Characteristics and outcome of dengue infection; clinical perspective from a secondary care hospital of Karachi. Pak J Med Sci. 2013; 29(1): 115–118. doi: 10.12669/pjms.291.2742 24353520 10.12669/pjms.291.2742 PMC3809171

