# Correction: The changing epidemiological pattern of Dengue in Swat, Khyber Pakhtunkhwa

**DOI:** 10.1371/journal.pone.0204465

**Published:** 2018-09-17

**Authors:** Jehangir Khan, Abdul Ghaffar, Shujaat Ali Khan

The following information is missing from the Funding section: This study was supported by the National Research and Development Plan of China (No.2016YFC1200500) and 111 project (B12003).

There is an error in the affiliation for the first author. Jehangir Khan is also affiliated with: Key Laboratory of Tropical Diseases and Control of the Ministry of Education, Guangzhou 510080, China.
